# Viral metagenomic survey of Caspian seals

**DOI:** 10.3389/fvets.2024.1461135

**Published:** 2024-09-18

**Authors:** Kobey Karamendin, Simon J. Goodman, Yermukhammet Kasymbekov, Marat Kumar, Sardor Nuralibekov, Aidyn Kydyrmanov

**Affiliations:** ^1^Laboratory of Viral Ecology, Research and Production Center for Microbiology and Virology, Department of Virology, Almaty, Kazakhstan; ^2^School of Biology, Faculty of Biological Sciences, University of Leeds, Leeds, United Kingdom

**Keywords:** Caspian seal, *Pusa caspica*, viral metagenome, marine mammals, pinniped, virome, wildlife diseases

## Abstract

**Introduction:**

Viral diseases of pinnipeds cause substantial mortality and morbidity and can influence population demography. Viral metagenomic studies can therefore play an important role in pinniped health assessments and disease surveillance relevant to both individual species and in a “One Health” context.

**Methods:**

This study used a metagenomic approach with high throughput sequencing to make the first assessment of viral diversity in Caspian seals (*Pusa caspica*), the only marine mammal species endemic to the Caspian Sea.

**Results:**

Sequencing libraries from 35 seals sampled 2009–2020 were analysed, finding sequences from the viral families *Circoviridae, Parvoviridae, Herpesviridae, Papillomaviridae, Picornaviridae, Caliciviridae, Cruciviridae, Anelloviridae, Smacoviridae,* and *Orthomyxoviridae,* with additional detection of *Adenoviridae* via PCR. The similarity of viral contigs from Caspian seal to sequences recovered from other pinnipeds ranged from 63.74% (San Miguel sea lion calicivirus) to 78.79% (Seal anellovirus 4).

**Discussion:**

Some findings represent novel viral species, but overall, the viral repertoire of Caspian seals is similar to available viromes from other pinnipeds. Among the sequences recovered were partial contigs for influenza B, representing only the second such molecular identification in marine mammals. This work provides a foundation for further studies of viral communities in Caspian seals, the diversity of viromes in pinnipeds more generally, and contributes data relevant for disease risk assessments in marine mammals.

## Introduction

Marine mammals are sentinels for ocean health (1). Monitoring the health status of wild marine mammal populations is essential for understanding the exposure and vulnerability of individual species to pathogens, the welfare of individuals and population dynamics, as well as the implications for the overall resilience of marine ecosystems to environmental change and anthropogenic threats (2, 3). Studies of marine mammal health are also relevant to the “One Health” context due to their interactions with humans and other species, and because anthropogenic activities may influence pathogen exposure risk. Long term monitoring suggests that in North America between 1991 and 2022, infectious diseases accounted for 14% of marine mammal deaths, with biotoxins (18%), ecological factors (14%), human interactions (5%), and other undetermined factors (49%) contributing the remainder (4). Infectious disease pathogens (viruses, bacteria and parasites), can influence individual mortality and morbidity, with consequences for survival and reproductive output, and drive mass mortality events, all of which can significantly impact marine mammal population demography (5). Therefore, it is important to develop baseline information about pathogen repertoires and prevalence to guide development of surveillance programmes, to assess temporal changes, and facilitate investigations of mass mortalities in support of marine mammal conservation and management (6).

Knowledge of the diversity of marine mammal pathogens is expanding rapidly (7–12), with morbilliviruses (5, 6) and influenza viruses (13, 14) being some of the best documented drivers of mass mortality events. The development of high throughput deep sequencing is driving new capability for pathogen discovery and surveillance beyond that achievable with standard serological and genetic approaches (15). In the context of viruses, metagenomic virome studies sequence the total nucleic acid profile from clinical or necropsy samples to assemble and identify sequence contigs from any pathogens present. Comparing recovered sequences to genomic databases, both known and novel viruses can be identified. Viromes for some members of the *Otariidae* family *(eared seals)* have been well characterized. Studies in California sea lions (*Zalophus californianus*) found previously undescribed RNA virus families for the species, including *Astroviridae*, *Picornaviridae*, *Caliciviridae*, and *Reoviridae*, and one DNA virus family (*Parvoviridae*) (16). An analysis of the virome of subantarctic fur seals (*Arctocephalus tropicalis*) and South American fur seals (*Arctocephalus australis*) has revealed the sequences of adenoviruses, circoviruses, picobirnaviruses, picornaviruses, herpesvirus-like viruses, anelloviruses, parvoviruses and one putative new genus, named *Nitorquevirus* (15, 17, 18). For phocid (earless) seals, metagenomic studies are less extensive, but have contributed to the discovery of novel anelloviruses, circoviruses, cycloviruses and papillomaviruses in Weddell seals (*Leptonchotes weddelli*) (19–22); papillomaviruses in Leopard seals (*Hydrurga leptonyx*) (21); and anelloviruses (23), varicellovirus (PHHV-1) (24), and gammaherpesvirus (PhHV-7) (25) in harbor seals (*Phoca vitulina*).

The Caspian seal (*Pusa caspica*) is a small-bodied phocid seal endemic to the Caspian Sea (26), and is classified as “Endangered” by the International Union for Conservation of Nature (IUCN) Red List, with a population size of around 168,000 (27). Historically, mass mortalities in Caspian seals were first recorded in 1955–56, when about 30 thousand animals died from a purported bacterial infection (28). However, at the time, the capacity to detect viral infections was limited, and the role of bacterial secondary infections under-appreciated. Further mass mortalities occurred in the Kazakh part of the Caspian Sea in 1968, 1971, 1978 and 1985, with the cause of death attributed to underwater blasting connected with hydro-engineering and military works (29), but with no toxicological, epidemiological and virological studies conducted. In 1997–2000 several large-scale seal stranding events were observed on the Caspian coast, with total mortality exceeding more than 11,000 seals (30). Canine distemper virus, a *Morbillivirus*, was identified as a primary causative agent (31). Since 2000, regular mass strandings have been reported ranging from 10s to 1,000s of individuals, but the causes remain poorly investigated due to challenging logistics and limited capacity for marine mammal pathology in the region (32, 33).

The Caspian seal, as a transboundary species, is a key indicator of the state of the Caspian ecosystem (34). In order to develop evidence-based conservation strategies it is important to be able to evaluate the effects of infectious diseases in the context of other anthropogenic factors such as impacts from shipping (35), fisheries bycatch (36), pollution (32), and historical hunting (37).

Kydyrmanov et al. recently reported on the long-term screening of prevalence for viruses, bacteria and parasites in Caspian seals, sampling 177 live, healthy, wild Caspian seals between 2007 and 2017, using serological and PCR based methods, concluding the overall pathogen repertoire and prevalence was comparable to other phocid seal populations (33). In that work, evidence for viral infections included CDV, phocine herpes virus, phocine adenovirus, influenza A, influenza B, and coronavirus. Here we report the first investigation of the virome of the Caspian seal using a metagenomic approach. The results add new knowledge on the viral epidemiology of this species, and will contribute to the understanding of current and future disease threats.

## Materials and methods

### Seal capture and sample collection

Samples for this study were collected during fieldwork in the Kazakh part of the northeastern and central Caspian Sea (Kazakh coastline and nearby islands) during spring and autumn of 2009, 2016, 2019 and 2020 (Supplementary Table S1). The seal haul out sites were reached by rigid inflatable boats. After stealthily approaching the seals on land, animals were caught using a “rush and grab” approach with hoop nets. Animals were restrained and handled without the use of chemical sedation, and were released immediately after the completion of sampling (33). Sampling was conducted in accordance with the Rules for conducting biomedical experiments, preclinical (non-clinical) and clinical studies (№ 697, 12 November 2007), Republic of Kazakhstan and Local Ethics Committee Research and Production Center for Microbiology and Virology (Approval number: 02-09-05 from 19 August 2014). In December 2020, about three hundred seals were reported stranded dead along the coast of Dagestan in Russia (38). Tissue samples (lung, spleen, kidney, large intestine) and swabs (tracheal, nasal, rectal) from eight carcasses from this event, stored in DNA/RNA shield were delivered to the laboratory and then archived at 4°C.

Body length (from nose tip to tail tip), girth and weight, and sex was recorded for each individual. Seals were categorised as juveniles (<1 year; body length 70–90 cm), sub-adults (older than 1 year of age; length > 91–109 cm), adults (mature, length > 110–140 cm). A complete external body examination was made for skin ulcers, parasites, trauma lesions and any visible alterations. Duplicate nasal, buccal, rectal, and urogenital samples were collected from captured seals using sterile cotton swabs, following Marine Mammal Commission guidelines (39). For virus isolation, swabs were placed in vials with viral transport medium (VTM) 199 containing antibiotics (penicillin 2000 U/mL, streptomycin 2 mg/mL, gentamicin 50 μg/mL, nystatin 50 U/mL) and bovine serum albumin at a final concentration of 0.5%. The samples were stored in liquid nitrogen (−196°C) until delivery to the laboratory. For molecular analysis, swabs were placed in RNA/DNA Shield reagent (Zymo Research, United States), which preserves viral nucleic acids at ambient temperatures.

### Sample processing

Before nucleic acid extraction, swab samples in VTM were centrifuged at 3200 RPM in an Eppendorf 5417R centrifuge (Rotor FA-45-24-11; Eppendorf, Hamburg, Germany) for 15 min and the resulting liquid was passed through a 0.22 μm filter (Membrane Solutions, United States) and then the filtrate was treated with a mix of nucleases: Benzonase (Sigma-Aldrich, United States), Turbo DNAse, DNAse I, RNAse A, and RNAse T1 (ThermoFisher, Lithuania). Solid tissue samples (5mm^3^) were placed in 2 mL vials with sterile PBS, pH 7.4 (Sigma Aldrich, United States) and beads, and homogenized with a TissueLyser instrument (Qiagen, Germany) shaking with a frequency of 25 cycles per second for 3 min, and centrifuged at 3200 RPM for 15 min to pellet the debris. Viral nucleic acids were then extracted from swab filtrates and tissue homogenate supernatants using the QIAamp Viral RNA Mini Kit (Qiagen, Germany) following the manufacturer’s recommendations. The QIASeq RNA Kit (Qiagen, Germany) was then used to generate double-stranded cDNA from input RNA.

### Sample pooling

Extractions from 35 seals were organised into 34 pools of different sample type combinations for sequencing library construction (Table 1; Supplementary Table S1). For material collected in Kazakhstan from 2016, 2019 and 2020, libraries consisted of either individual or pooled samples. Within years, extractions from the same sample type (buccal, nasal, genital, rectal and conjunctival swabs) were pooled by sex-age class (juvenile males, adult males, juvenile females, and adult females). Additionally, one library was constructed from a buccal swab from an adult female sampled in 2009, which was PCR-positive for avian influenza A (33). Lastly, a library was produced for a necropsy spleen sample from one of the Dagestan seals from the 2020 mass mortality event.

**Table 1:** Year/sex/age class composition of sample pools and total contig counts for each detected virus family found in each pool.

Sample ID	Age	Sex	Sample Type	Total contigs of viral families found									
				Circo-	Herpes-	Parvo-	Papillo-	Picorna-	Calici-	Anello-	Smaco-	Orthomyxo-	Cruci-
Seal-Flu-2009-11*	Ad	F	B									5	
Seal_2016–3	Ad	M	N	23	15								
Seal_2016-4B	Ad	F	B	1				3					
Seal_2016-4G	Ad	F	G	2				14					
Seal_2016-4 N	Ad	F	N					2					
Seal_2016-4R	Ad	F	R	16				82					
Seal_2016-5G	Ad	F	G	2									
Seal_2016-6R	Ad	F	R					2					
Seal_2016–16R	Ad	F	R					6					
Seal_2019-pool-1	Ad	M	B		15				11				
Seal_2019-pool-2	Juv	F	B		22								
Seal_2019-pool-3	Ad	F	B		10								
Seal_2019-pool-4	Ad	M	G		25								
Seal_2019-pool-5	Juv	F	G		5								
Seal_2019-pool-6	Ad	F	G		30				26				
Seal_2019-pool-7	Ad	M	R		10				17				
Seal_2019-pool-8	Juv	F	R		12								
Seal_2019-pool-9	Ad	F	R		393				33				
Seal_2020-pool-1	Juv	M	C + B	84	6	14	1						
Seal_2020-pool-2	Juv	M	N	32		10							5
Seal_2020-pool-3	Juv	M	G	405		14							15
Seal_2020-pool-4	Juv	M	R	121		852						1	29
Seal_2020-pool-5	Ad	M	C + B	132	5	6	248						
Seal_2020-pool-6	Ad	M	N	16		1	17						
Seal_2020-pool-7	Ad	M	G	99			1			29	10		
Seal_2020-pool-8	Ad	M	R	18			15						
Seal_2020-pool-9	Juv	F	C + B	19		8	1						
Seal_2020-pool-10	Juv	F	N	268		22	4						
Seal_2020-pool-11	Juv	F	G	289		1							
Seal_2020-pool-12	Juv	F	R	50						11		1	
Seal_2020-pool-13	Ad	F	C + B	75			3						
Seal_2020-pool-14	Ad	F	N		8		34						
Seal_2020-pool-16	Ad	F	R	293		251							
Seal_2020-Dagestan*	Ad	F	SP				1					1	
Total Contigs				1945	556	1,179	325	109	87	40	10	8	49

* Sample from dead animal; Age: Ad – Adult, Juv – juvenile; Sex: F – female, M – male; Sample Type: B – buccal, N – nasal, G – genital, R – rectal, C – conjunctival, SP – spleen.

### Metagenomic sequencing and bioinformatics

For massively parallel sequencing, libraries were constructed using the NEBNext Ultra DNA Library Preparation kit (New England Biolabs, United States) according to the manufacturer’s protocol. Library size selection was performed using Ampure XP beads (Beckman Coulter, United States). The size and quality of libraries were checked on a Bioanalyzer 2,100 instrument (Agilent Technologies, Germany). Sequencing was performed using the MiSeq Reagent version 3 kit on a MiSeq sequencer (Illumina, United States), at the Research and Production Center for Microbiology and Virology, Almaty, Kazakhstan. The reads were trimmed, and their quality was assessed with FastQC (40). Sequence reads passing quality control filters were *de novo* assembled using Geneious 11.0 software (Biomatters, New Zealand) (41) by applying the installed SPAdes assembler (42) with default parameters. The resulting contigs were subjected to BLASTn and BLASTx searches in the local viral reference database as described in the Metavisitor pipeline (43). Local BLAST hits with lengths >200 nucleotides (nt) were considered significant at E value <10^e-5^, and the potential viral sequences were subjected to a new BLASTx search against non-redundant protein sequences (nr) from the NCBI database. The best-hit sequences were individually annotated to the matching viral sequences. The viral sequences were verified by mapping reads to the corresponding reference genomes in the Geneious 11.0 software (Biomatters, New Zealand).

### PCR confirmation of viral detections

Following identification of viral contigs in the metagenomic analysis, PCR was carried out with primer sets synthesized for each viral species to confirm the presence of virus-specific nucleic acids in samples. PCR was undertaken using an Eppendorf Gradient amplifier using appropriate thermal cycling conditions. The presence of viral sequences was verified using PCR/RT-PCR on the original RNA/DNA samples with corresponding primer sets (44–48).

### Phylogenetic analysis of metagenomic sequences and assessment of heterogeneity in virome diversity

Phylogenetic analysis of viral contigs derived from metagenomic sequencing, together with appropriate representative reference sequences, was carried out using the neighbor-joining method with 1,000 bootstrap replicates using the p-distance in MEGA X (49).

Variation in sample viral diversity in relation to sample type, year, and sex-age class was visualized using a heatmap and cluster analysis implemented using the ComplexHeatmap package (50) in RStudio (v. 2022.12.0 + 353). The heatmap colour-scale illustrates the virome diversity quantified by the number of contigs observed for each virus family within a particular pool/sample. A hierarchical cluster analysis was conducted in relation to sample type, by calculating a Euclidean distance based on viral abundances in each sample/pool, and then using the resulting distance matrix to cluster according the complete linkage algorithm.

## Results

### Metagenomic sequencing, BLAST search, and viral family relative abundance

An average of approximately 1,250,000 raw sequencing reads per library were obtained. Following assembly of contigs, BLAST searches revealed the presence of viruses belonging to 10 families, with viral contig counts ranging from 0 to 405 for each virus family-library combination (Table 1). Viral contigs ranged in size from 71 to 790 nt, and showed sequence identity ranging from 58 to 98.23% with known viruses (Table 2), suggesting some of these sequences might be derived from novel viral species. Contigs up to approximately 1 kilobase in size were found with partial homology to *Circoviridae, Picornaviridae and Caliciviridae* families but we retained for analysis only those parts that could be reliably aligned with known sequences from GenBank to avoid to avoid potentially ambiguous results.

**Table 2:** The most significant BLASTx hits matching known eukaryotic viruses, for contigs, obtained from Caspian seal samples.

Family/Genus	Accession number	Contig length (nt)	Genome	Product	Best hit	Amino acid identity (%)	E-value	(RT)-PCR Confirmation
*Circoviridae*	PP744471	207	ssDNA	Replication initiation protein	Replication initiation protein [Humpback whale blow-associated circo-like virus 2] (AWR89666)	78.26	2e-29	No
*Parvoviridae*	PP744472	175	dsDNA	NS2 protein	NS2 protein [uncultured sea star-associated densovirus] (QOD39598)	94.64	8e-28	No
*Parvoviridae*	PP744473	144	ssDNA	Capsid protein VP1	Capsid protein VP1 [Canine parvovirus] (QBO24456)	58.33	6e-09	No
*Herpesviridae*	PP744474	312	dsDNA	EUL25 protein	Protein EUL25 [Equid alphaherpesvirus 1] (AII80971)	81.33	1e-29	Yes
*Papillomaviridae*	PP744475	186	dsDNA	E2 Protein	E2 protein [*Leptonychotes weddellii* papillomavirus 6] (NC_040818)	72.58	2e-20	No
*Picornaviridae*	PP744476	790	+ssRNA	Polyprotein	Polyprotein [Racoon dog picornavirus] (UMO75562)	72.59	2e-128	No
*Caliciviridae*	PP744477	273	+ssRNA	ORF-1	ORF-1 protein [San Miguel sea lion virus 8] (AKG26793)	63.74	5e-31	No
*Anelloviridae*	PP744478	102	ssDNA	ORF1	ORF1 [Seal anellovirus 4] (YP_009115496.1)	78.79	3e-09	No
*Smacoviridae*	PP744479	96	ssDNA	Eplication associated protein	Capsid protein [Swine associated smacovirus] (QCC72666)	71.88	1e-04	No
*Cruciviridae*	–	183	ssDNA	REP	Putative replication associated protein [Crucivirus sp.] (QKV51008)	45.59	4e-08	No
*Orthomyxoviridae/Influenza A*	PP744480	71	-ssRNA	Hemagglutinin gene	Hemagglutinin gene (Influenza A virus A/*Anas platyrhynchos*/Belgium/3950–8/2015(H3N8)) [MT406985]	95.83	1e-21	Yes
*Orthomyxoviridae/Influenza A*	PP744481	276	-ssRNA	Nonstructural protein	Nonstructural protein 1 (NS1) gene [Influenza A virus A/Blue-winged Teal/Kansas/AH0029699S.8.B/2015 (H12N6)] (QDX48107)	92.94	3e-48	Yes
*Orthomyxoviridae/Influenza B*	PP744482	312	-ssRNA	Polymerase PB2	Polymerase PB2 [Influenza B virus (B/Florida/09/2016)] (APW80077)	97.12	4e-59	Yes
*Orthomyxoviridae/Influenza B*	PP744483	360	-ssRNA	NS protein	NS protein [Influenza B virus (B/Arizona/38/2016)] (AQS98133)	98.23	7e-75	Yes

**Figure 1:**
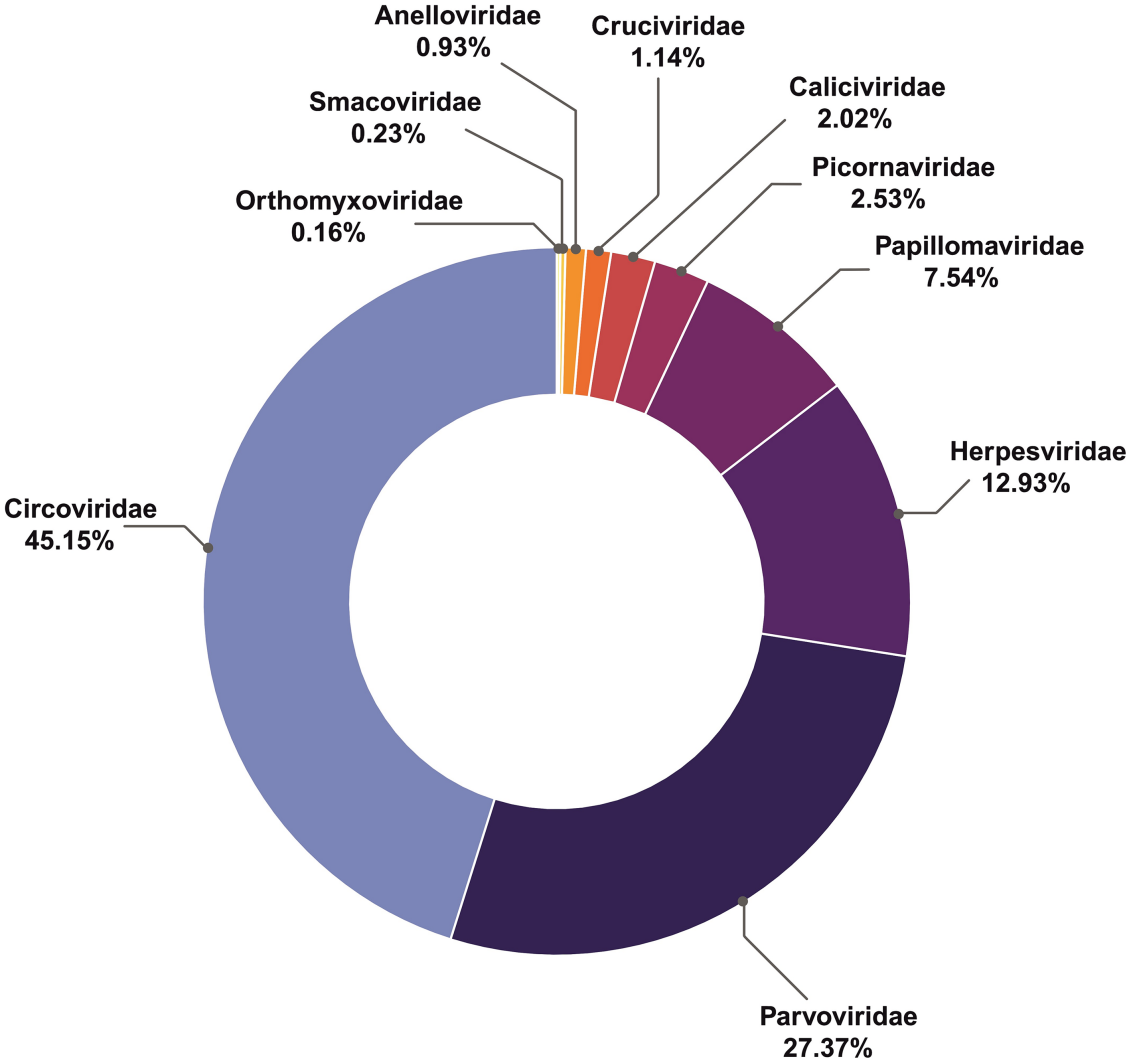
Proportions of viral families in across all samples.

Overall, the most abundant viral contigs (Figure 1), comprising 45% of those recovered, matched the family *Circoviridae*. More than a quarter (27.3%) were assigned to parvoviruses, (12.9%) matched herpesviruses, followed by *Papillomaviridae* (7.5%), *Picornaviridae* (2.5%), *Caliciviridae* (2%), *Anelloviridae* and *Cruciviridae* (both approximately 1%). The rarest contigs belonged to *Smacoviridae* (0.23%) and *Orthomyxoviridae* (0.16%).

### PCR confirmation

Of the 10 viral families found in metagenomic sequencing, confirmatory PCR amplicons were recovered only for viruses from *Herpesviridae* and *Orthomyxoviridae*. Additionally, although no *Adenovirus* contigs were recovered in the metagenomic sequencing, PCR-screening detected adenovirus products from buccal, nasal and rectal swabs from six live seals sampled in 2020, adding to PCR detections of adenovirus in samples from earlier years reported in our previous study (33).

Previously we reported molecular and serological evidence for ongoing circulation of Canine morbillivirus (formerly canine distemper virus; CDV), a member the family *Paramyxoviridae*, in Caspian seals (33). In this research, no CDV contigs were detected for metagenomic sequencing of samples collected 2009–2020, and RT-PCR testing for CDV in 2019–2020 samples was also negative.

### Heterogeneity in sample virome diversity

**Figure 2:**
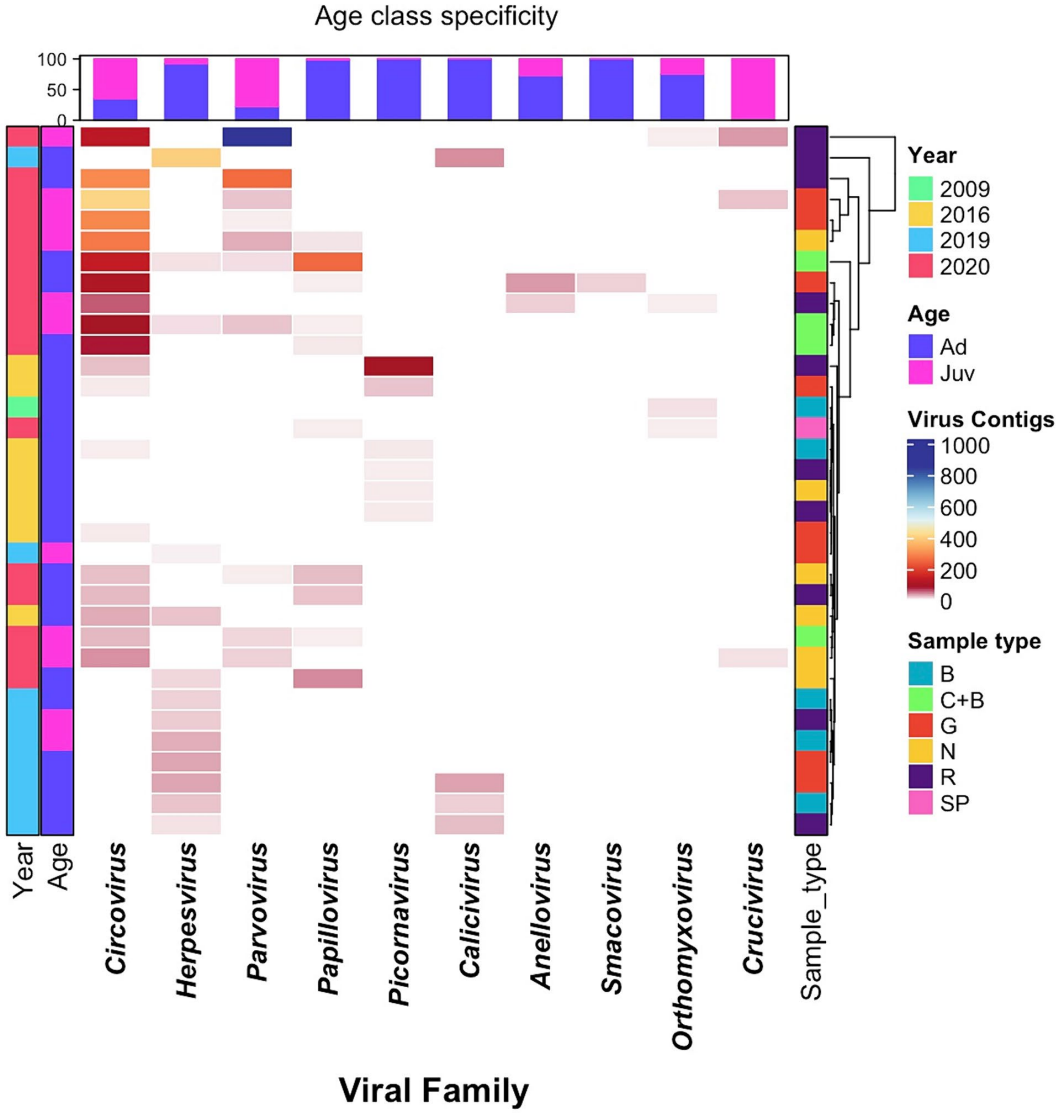
Heatmap of virome diversity of seals, based on age and sample types across different years, illustrating virus relative abundance profiles, with each cell representing the contig counts belonging to the given virus family, indicated by corresponding colors. Samples are from dead seals; Age: Ad – Adult, Juv – Juvenile; Sample Type: B – Buccal, N – Nasal, G – Genital, R – Rectal, C – Conjunctival, SP – Spleen.

Analysis of the age-specific virus patterns revealed a notable disparity in the prevalence of circovirus and parvovirus contigs, with juvenile seals exhibiting a higher abundance compared to adults, a trend that was particularly pronounced in 2020. Additionally, the results highlight the high prevalence of herpesvirus among adult seals in 2019. *Parvoviridae, Papillomaviridae, Anelloviridae, Smacoviridae,* and *Cruciviridae* families were found only in samples from 2020, with the *Caliciviridae* family found only in 2019, and *Picornaviridae* present only in 2016. The majority of the *Herpesviridae* sequences were recovered from 2019 samples, but were also found at low frequency in 2016 and 2020. *Circoviridae* contigs were overwhelmingly found in 2020, had smaller numbers 2016, and were absent in 2019 (Table 1; Figure 2).

### Description of identified viral contigs

*Circoviridae* was the most abundant family found. A single contig type of 207 nt was recovered, matching the replication initiation protein from humpback whale blow-associated Circo-like virus 2, discovered in Australia in 2017 (51), with a BLASTx amino acid similarity of 78.26%. Phylogenetic analysis (Supplementary Figure S1A) supported the same association.

The second most abundant family was *Parvoviridae.* Two distinct parvovirus contigs were found. The first, matching Parvovirus-like VP1 gene sequences, was 58.33% similar to Canine parvovirus in BLASTx searches. The second, matching Parvovirus-like nonstructural NS2 gene sequences, was 94.64% similar to densoviruses found in sea star, invertebrates, insects, and decapod crustaceans (52). Phylogenetic analyses with the partial NS2 protein sequence (Supplementary Figure S1B) suggest they are associated with parvoviruses from other aquatic organisms, clustering with densoviruses from oyster, mollusks, and sea stars.

*Herpesviridae* contigs were recovered for a fragment of the EUL25-like protein gene with 81.33% similarity to Equid alphaherpesvirus 1 in BLASTx analysis, which formed a separate cluster from the corresponding Phocid herpesvirus 1 cluster in a phylogenetic tree (Supplementary Figure S1C).

The *Papillomavirus* contig sequence found in Caspian seals was 72.58% similar to the E2 protein sequence of papillomavirus 6 from Weddell seals in BLASTx searches (22). Phylogenetically, the Caspian seal papillomavirus clusters most closely with other mammalian Papillomaviruses from rodents, mustelids and cats (Supplementary Figure S1D).

We recovered a Caspian seal *Picornavirus* contig of 790 nt with 72.59% similarity in BLASTx analysis to a Racoon dog picornavirus polyprotein gene protein isolated in China in 2017–2021. Phylogenetic analysis suggested that Caspian seal picornavirus was taxonomically closest to California sea lion sapelovirus, with both forming a clade as part of a lineage also containing Racoon dog picornavirus, and Feline picornavirus (Supplementary Figure S1E).

In BLASTx searches, a 273 nt contig for vesivirus-like sequences found in this study were 63.74% similar to the ORF-1 protein sequence from San Miguel sea lion virus 8, isolated in the USA (53). The vesivirus genus is a lineage of the *Caliciviridae* family. Phylogenetic analyses confirmed its relatedness to the San Miguel sea lion virus and canine vesiviruses (Supplementary Figure S1F).

The Caspian seal anellovirus sequence, represented by a 102 nt contig, was 78.79% similar in BLASTx search to Seal anellovirus 4 ORF-1 protein (25). Phylogenetic analysis of the partial ORF1 nucleotide sequence of Caspian seal anellovirus showed its relatedness to a cluster consisting of Seal anelloviruses 4 and 5 (Supplementary Figure S1G).

*Smacoviridae and Cruciviridae* family sequences were found in samples from 2020. In BLASTx searches, the Caspian seal 96 nt smacovirus contig sequence was 71.88% similar to Swine associated smacovirus capsid protein. The putative crucivirus 189 nt sequence in BLASTx search was 45.59% similar to replication associated protein from crucivirus found during metagenomic data mining of environmental water (54).

For *Orthomyxoviruses*, sequences from the hemagglutinin and nonstructural protein genes of Influenza A virus were recovered from one of the 2009 samples (Table 2). BLASTx searches revealed they were 95.8 and 92.94% similar to influenza virus sequences of H3 and H12 subtypes isolated from wild birds in 2006–2019 worldwide. Phylogenetic analysis of the H3 hemagglutinin and non-structural protein genes confirmed the relatedness of the Caspian influenza A sequences to the avian Eurasian lineage (Supplementary Figures S1H,I).

Two distinct influenza B virus contigs were identified, for the first time in Caspian seals, in the spleen sample from the dead animal stranded in Dagestan (Russia) in 2020. One sequence matched the PB2 polymerase gene, showing 97.12% similarity with the B/Florida/09/2016 virus isolated from humans. The second from the nonstructural NS gene, showed 98.23% similarity with the B/Arizona/38/2016 virus. Phylogenetic analyses returned a similar pattern of clustering (Supplementary Figures S1J,K).

## Discussion

In this study, we made the first evaluation of viral diversity of Caspian seals using a metagenomic approach, recovering viral contigs belonging to families whose representatives are linked to both apathogenic or disease-causing infections in mammals. The most abundant contigs observed in samples belonged to the *Circoviridae* family, which are small non-enveloped circular single-stranded DNA viruses. Pathogenic circovirus infections are found in porcine and avian species worldwide. Subclinical infections are more common, but porcine circovirus 2, infectious chicken anemia, and psittacine beak and feather disease are associated with severe disease courses (55). The Caspian seal circovirus was 78.26% similar to the recently identified novel circovirus-like virus detected in Humpback whale blow (51). These circo-like viruses appear to be significant virome components in many marine environments and marine invertebrates may be the primary host (56). For now, the biological and clinical significance of these novel circovirus for marine mammals remains unknown.

*Parvoviridae*, which are non-enveloped linear ssDNA viruses, was the second most abundant family in tested samples. We found contigs with 58.3% similarity to canine parvovirus, 94.64% similarity to marine invertebrate denosviruses, respectively. Densoviruses are known to infect invertebrates, insects, and decapod crustaceans (52). Thus, these parvovirus-like sequences found appear related to parvoviruses associated with aquatic ecosystems. These findings parallel those for a study of the humpback whale blow virome where sequences from *Circoviridae* and *Parvoviridae* families also formed the top two most abundant read classes (51). Further work will be needed to confirm the number of strains and taxonomy of these viruses in Caspian seals.

*Herpesviridae* is a large family of enveloped dsDNA viruses (1), with isolates from Pinnipeds first identified in Atlantic harbor seals in 1985 (57). To date seven phocid herpesviruses have been recorded worldwide – phocid herpesviruses from type-1 to type-7 (PhHV 1–7) (25). Newborn and immunosuppressed seals infected with PhHV-1 die with symptoms of acute pneumonia and focal hepatitis but adults show no apparent infection (46). PhHV-2 is more distantly related to both PhHV-1 and herpesviruses of terrestrial carnivores (46). Previously, we found a herpesvirus Us2-like protein partial sequence in Caspian seals (33) that was 100% similar to Phocid alphaherpesvirus 1 from Atlantic harbor seal (58). In the current study we recovered partial contigs for the EUL25 protein with 81.33% similarity to Equid alphaherpesvirus 1. Further studies are necessary to obtain the complete genome of the Phocid alphaherpesvirus 1 from Caspian seals for full characterization, and to determine the number of strains. If PhHV-1 from Caspian seal behaves similarly to other alphaherpesviruses, it may establish latency in neurons (59), without manifesting any clinical signs, but additional work is needed to determine infection mechanisms.

*Papillomaviruses* are small, non-enveloped, circular dsDNA viruses that infect skin or mucosal membranes in a wide range of animals, including marine mammals (60). In marine mammals, *Papillomaviridae* family viruses have been found in manatees (61), cetaceans (62) and pinnipeds (63). Clinical signs include warty lesions in lingual and genital mucosa and cutaneous tissues. Some papillomavirus species have been identified as oncogenic (59). Papillomavirus E2 protein contig sequences from Caspian seals were 72.58% similar to *Leptonychotes weddellii* papillomavirus 6, but proliferative axillary lesions were not visible in the Caspian seals examined.

The *Picornaviridae* family comprises small, non-enveloped, positive sense ssRNA viruses. Sequences from Caspian seals were 72.59% similar to Racoon dog picornavirus isolated from healthy animals in China in 2021 (64). However, phylogenetic analysis indicated that Caspian seal picornavirus was clustered closest to California sea lion sapelovirus in a lineage that also include the Racoon dog virus. Raccoon dogs are common around the Northern Caspian seashore and frequently visit haul-out sites of Caspian seals. Thus, conceivably, this picornavirus species could circulate in both the local carnivore and Caspian seal population but further surveys of terrestrial carnivores are required to determine the extent of shared viral repertoires among the taxa. Picornaviruses cause notable veterinary diseases with significant mortality, including foot-and-mouth disease in hoofed animals and avian encephalomyelitis in poultry. In marine mammals, picornaviruses have been found in sub-Antarctic fur seals, South American fur seals (18), California sea lions (16), harbor seals (65), ribbon and ringed seals (66), but their role in pathology still needs to be determined.

*Caliciviridae* is family of non-enveloped single-stranded, positive sense RNA viruses. Marine caliciviruses are associated with the *Vesivirus* genus (67), and it has been suggested that marine mammals serve as their natural reservoir (68). Sequences were detected in samples from Caspian seals from fragments of nonstructural polyprotein gene sequences that were 63.74% similar to San Miguel sea lion virus (SMSV), identified in the USA in 1972. This San Miguel sea lion virus belongs to a distinct lineage within the genus *Vesivirus*, which also includes Vesicular Exanthema of Swine Virus (VESV). VESV is clinically indistinguishable from picornaviral vesicular diseases such as foot-and-mouth disease, swine vesicular disease and rhabdoviral vesicular stomatitis. It has been suggested that VESV, SMSV, and other related viruses originated from marine mammal caliciviruses (53), with marine vesiviruses circulating worldwide. Clinically, SMSV causes vesicular lesions on the flippers and the mouth, as well as gastroenteritis in California sea lions (53). No lesions were observed in Caspian seals at the time of sampling, although the overlooking of small lesions cannot be ruled out.

*Anelloviridae* sequences detected in Caspian seals were 78.79% similar to ORF1 of Seal anellovirus 4 identified from harbor seals (*Phoca vitulina*) in the Netherlands in 2014. Anelloviruses are non-enveloped, circular ssDNA viruses (18). The role of anelloviruses in the pathology of wild animals, including pinnipeds, is not well understood, although many viruses have been genetically characterized. For human anelloviruses, a clear link between anellovirus positivity and disease has not been established (69), but recent studies have suggested that some anelloviruses are associated with unexplained fever, diabetes, cirrhosis in liver transplant patients, respiratory disease, cancer, and autoimmune disorders (70).

Influenza viruses of the *Orthomyxoviridae* family are also significant for the health status of marine mammals. *Orthomyxoviruses* are enveloped viruses with a segmented RNA genome. Diseases caused by influenza viruses of avian origin from subtypes H7N7 (71), H4N5 (72), H3N3 (73) and H13 (74) subtypes are known in pinnipeds. A pandemic pdmH1N1 strain (75), that spread globally in 2009 has also been isolated from healthy-looking seals, which indicates the potential of seals to serve as a reservoir of influenza viruses in the wild. Additionally, H5N1 influenza A has recently spilled over into pinnipeds in both the northern and southern hemispheres (13, 14, 76, 77) and in Antarctica (78) causing mass mortalities.

Previously, a H4 influenza A subtype was described in Caspian seals (79). In this study, we make the first report of sequences from the H3 influenza A hemagglutinin gene to be detected from Caspian seals as well as the nonstructural protein. This hemagglutinin sequence was 95.83% similar to H3 subtype influenza viruses of the Eurasian lineage isolated from wild birds. This subtype was also previously found in pinnipeds (73). The sequence was derived from a sample collected in 2009 from a live, apparently healthy individual. Antibodies to the influenza A virus have previously been found in the Caspian seal (33, 80) with RT-PCR confirmation (33), but to date influenza A has not been linked conclusively to significant mortality events in Caspian seals. Analysis of marine mammal influenza viruses suggests they were acquired through close contact with wild birds such as oral–fecal transmission through seawater (72). In the Caspian region, sea and migratory birds regularly share seal haul out sites, creating opportunities for this route of transmission (33, 81). Scavenging of carcasses of dead birds by Caspian seals has not been observed or reported in dietary studies (26, 76, 82), so infection with influenza viruses through this route, as has been suggested for some recent H5N1 transmissions elsewhere (83), may be less likely.

Influenza B virus sequences were also identified in Caspian seals. Influenza B viruses were first isolated from a juvenile harbor seal in a rehabilitation center in the Netherlands (47). It was suggested that the virus had been introduced in the seal population from a human source and circulated in seals for some time. Further, antibodies to influenza B were detected in harbor and grey seals in the 2010s (84), also in Caspian seals (33, 61). In this study, influenza B sequences were found in a spleen sample from a dead seal that stranded on the in the Dagestan seashore during a mass mortality event in 2020 (38). BLAST search has shown that the partial sequences of the Caspian seal influenza B virus PB2 and NS genes were closely related to those of strains circulating in humans simultaneously in 2020 and 5 years earlier (data not shown). However, this finding in isolation is not sufficient to attribute influenza B as factor in the mass mortality. At this time there is not sufficient data to consider whether seals are a reservoir of influenza B in nature, but we can see that seals are also susceptible to this virus and the routes of seal exposure are still to be determined.

Both *Smacoviridae and Cruciviridae* possess small circular single-stranded DNA genomes. S*macoviridae* is frequently found in vertebrate species, but not in environmental samples, and was previously known as “stool-associated circular virus” detected in healthy and diarrheic mammals, birds as well as in insect species (85), with one previous report in sea lions (10). *Cruciviridae* possess a chimeric genome with genes derived from eukaryotic ssRNA and ssDNA viruses (86). *Smacoviridae and Cruciviridae* have not been studied in marine mammals and their evolutionary and ecological history in this context is unclear. At this time, we can only state their presence in the viral metagenome of the Caspian seal, and further research is needed to deepen our knowledge of these viral families.

*Adenoviridae* family viruses are double-stranded DNA viruses that infect all groups of vertebrates, including marine mammals. Canine adenovirus 1 causes viral hepatitis in dogs and infects many wildlife species (87). Adenoviruses are known to cause fatal hepatitis in different otariid species (45, 88). In our previous research, we have identified an adenovirus sequence that is 76% (33) similar to Phocine adenovirus 1 from a Northern elephant seal (*Mirounga angustirostris*) with ocular lesions (89). It was suggested that the presence of this virus in ocular tissues of pinnipeds was common and not significantly associated with the disease (89). Here we did not detect adenoviral sequences from metagenomic sequencing, but PCR screening revealed positive samples collected from live seals in 2020, adding to those from our earlier study. This suggests that direct PCR detection may be more sensitive than NGS based approaches when testing swab samples from seals. Elsewhere, adenoviruses in pinnipeds have also mainly been detected via PCR (45, 88–90). To our knowledge, adenoviruses have not been found in other metagenomic studies of pinniped viromes (10, 15, 16, 18, 24). It is possible that methodological bias in isolation, extraction, and/or sequencing impedes detection of adenoviruses in NGS studies, or that pinniped adenoviruses are genetically distant from known strains, meaning they do not return robust matches in BLAST searches. However, this remains to be resolved in further work.

Representatives of the genus *Morbillivirus* of the *Paramyxoviridae* family are some of the most important pathogens for marine mammals. CDV has previously caused mass mortalities of thousands of animals in Caspian and Baikal seals (*Pusa sibirica*) (31, 91). Our previous work detected antibodies to CDV in Caspian seals with ELISA tests for six sera out of 74 (8.1%) animals sampled from 2007 to 2017, and 5 of 13 asymptomatic animals sampled in 2008 were positive for PCR tests (33). Two complete CDV genomes were recovered for the 2008 animals, which formed a clade with 99.59% identity to the 2000 Caspian seal epizootic lineage (33). In the current work, no morbillivirus contigs were recovered from metagenomic sequencing of samples 2009–2020, and the were no positive detections by RT-PCR for 2019–2020 samples. Together, our two studies and others (92), suggest that CDV has circulated episodically in Caspian seals since 2001, but the trigger mechanisms for mass mortalities are still unknown.

Our study has some limitations. Firstly, only short viral sequences were identified in the metagenomic sequencing with a maximum contig size of 790 nt. This size range is comparable to that found in other studies. For example, Kluge et al. (18) described the virome of subantarctic and south American fur seals, recovering contigs ranging from 227 to 1,519 nt, but in other studies multi-kilobase scale contigs has been reported (10, 16). Small contig sizes could be due to low viral load in samples which would diminish the recovery of unfragmented nucleic acids after viral enrichment and extraction steps. Most of our samples came from clinically healthy animals, and for necropsy material there could be some post-mortem degradation, both of which could contribute to low recoveries of viral sequences. Low homology with reference sequences can also reduce contig assembly success. However, given, that overall, our results on viral family presence appear comparable with other pinniped virome studies, it suggests that we have been able to successful detect core virome constituents, but it is possible that further viral taxa would be identified with more extensive sampling and deeper sequencing. The short sequence lengths also mean that the phylogenetic position of the viruses requires further confirmation, but this is also restricted by reference sequence coverage for most of the families, and the extent of sequence homology. Improving sequencing yields in future work to recover full viral genomes will greatly enhance the capacity to determine viral identity, phylogenetic relationships and evaluations of disease potential, and viral functional diversity. Secondly, it was not possible to independently confirm viral presence via PCR/RT-PCR in all cases due to lack of reliable conserved primers for all families, which limited amplification success. Expanding genomic coverage for these viruses will support future primer development for use in diagnostic and confirmatory testing. Lastly, inferring viral abundance from contig read counts is an indirect indicator, and should be interpreted cautiously, but is commonly taken as representative of relative prevalence in metagenomic studies (15, 16, 18).

In conclusion, the viral metagenomic profile of Caspian seals is similar to those from other marine mammals and all viral families described in this research have previously been reported in marine mammals. The two most dominant viral families consisting of *Circoviridae* and *Parvoviridae* families found in this study are widespread in marine ecosystems. The primary hosts of these viruses are likely to be Caspian Sea marine invertebrates in the case of circoviruses, or insects and crustaceans in the case of densoviruses in the *Parvoviridae* family. These two families make up 72% of the entire Caspian seal virome, and are possibly of dietary origin, since Caspian seals frequently feed on crustaceans and invertebrate consuming fish (82).

A second major group in the virome comprises mammalian viruses: *Herpesviridae, Papillomaviridae, Caliciviridae, Anelloviridae, Adenoviridae, Orthomyxoviridae* and *Paramyxoviridae*. Viruses of this group can potentially cause various pathologies in mammals or be asymptomatic (93). It is still unclear to which group the *Picornaviridae*-like sequences belong since the identified contigs are genetically distant from mammalian and aquatic viruses.

In terms of pathogenicity, some viral species from the *Circoviridae* and *Parvoviridae* families are pathogenic for mammals, but the species identified in the Caspian seal appear to originate with marine invertebrates, and therefore may present a low disease risk. Among the mammal associated viruses, the most pathogenic are the *Orthomyxoviridae* (influenza A subtype H5) and *Paramyxoviridae* (Morbilliviruses) families, which are capable of causing mass mortalities, but the available data suggests that these mostly circulate as low virulence strains in healthy seals during interepizootic periods. In future work, high priority should be given to identifying potential reservoirs and zoonotic transmission routes for these viral taxa, and the environmental, genetic, ecological, and epidemiological triggers for mass mortality events. *Herpesviridae, Papillomaviridae, Caliciviridae* and *Adenoviridae* are capable of causing clinical manifestations of varying severity in pinnipeds or asymptomatic infections. In the Caspian seal they were detected in clinically healthy seals, and further research is needed to deepen our knowledge of their viral pathology. The role of viruses of the *Anelloviridae*, *Smacoviridae* and *Cruciviridae* families in infectious pathology is still unclear and further research is also needed.

The similarity of Caspian seal viruses with counterparts in other animals worldwide varied from 45 to 98%. The habitat of the Caspian seal is restricted to the landlocked Caspian Sea, with no direct access to the World Ocean. The Caspian Sea is a remnant of the Paratethys, and is last thought to have shared an open connection the world’s ocean 35 million years ago, although sporadic connections with the Arctic Ocean and Mediterranean Sea likely existed through Pleistocene glacial cycles (94, 95). Caspian seals are estimated to have diverged from sister taxa around 1 to 2 million years ago (96). Therefore, given this history of environmental and species isolation, the discovery of genetically distant viruses was expected. As virome data from marine mammals accumulates, results such as these can contribute to comparative studies of the ecology and evolution of pathogen communities and their implications for marine mammal health. These results will contribute to new resources and strategies for disease surveillance in Caspian seals. Given the regular occurrence of mass mortalities, with annual strandings of hundreds to thousands of carcasses, it is essential to develop capacity for pathology investigations to support integrated Caspian seal conservation (33), with particular priority given to coordinated transboundary surveillance for influenza and CDV.

## Data Availability

The datasets presented in this study can be found in online repositories. The names of the repository/repositories and accession number(s) can be found in the article/Supplementary material.

## References

[1] Gulland F.M.D. Dierauf L.A. Whitman K.L., CRC handbook of marine mammal medicine. Third edition. ed. (2018), Boca Raton: CRC Press, Taylor & Francis Group. xix–1124 pages.

[2] Barratclough A Ferguson SH Lydersen C Thomas PO Kovacs KM. A review of circumpolar Arctic marine mammal health-a call to action in a time of rapid environmental change. Pathogens. (2023) 12:937. doi: 10.3390/pathogens12070937, PMID: 37513784 PMC10385039

[3] Simeone CA Gulland FMD Norris T Rowles TK. A systematic review of changes in marine mammal health in North America, 1972–2012: the need for a novel integrated approach. PLoS One. (2015) 10:e0142105. doi: 10.1371/journal.pone.0142105, PMID: 26579715 PMC4651562

[4] Anonymous. National Oceanic and Atmospheric Administration fisheries. Marine Mammal Protection Marine Mammal Unusual Mortality Events. (2024); Available at: https://www.fisheries.noaa.gov/national/marine-mammal-protection/marine-mammal-unusual-mortality-events (Accessed September 03, 2024).

[5] Harkonen T Dietz R Reijnders P Teilmann J Harding K Hall A. The 1988 and 2002 phocine distemper virus epidemics in European harbour seals. Dis Aquat Org. (2006) 68:115–30. doi: 10.3354/dao068115, PMID: 16532603

[6] Duignan PJ Van Bressem MF Baker JD Barbieri M Colegrove KM De Guise S. Phocine distemper virus: current knowledge and future directions. Viruses. (2014) 6:5093–134. doi: 10.3390/v6125093, PMID: 25533658 PMC4276944

[7] Nollens HH Wellehan JFX Archer L Lowenstine LJ Gullande FMD. Detection of a respiratory coronavirus from tissues archived during a pneumonia epizootic in free-ranging Pacific harbor seals Phoca vitulina richardsii. Dis Aquat Org. (2010) 90:113–20. doi: 10.3354/dao02190, PMID: 20662367

[8] Palacios G Wellehan JFX Raverty S Bussetti AV Hui J Savji N. Discovery of an orthoreovirus in the aborted fetus of a Steller Sea lion (Eumetopias jubatus). J Gen Virol. (2011) 92:2558–65. doi: 10.1099/vir.0.032649-0, PMID: 21795475 PMC3352366

[9] Wellehan JF Jr Rivera R Archer LL Benham C Muller JK Colegrove KM. Characterization of California Sea lion polyomavirus 1: expansion of the known host range of the Polyomaviridae to Carnivora. Infect Genet Evol. (2011) 11:987–96. doi: 10.1016/j.meegid.2011.03.010, PMID: 21453794

[10] Vigil K Aw TG. Comparison of de novo assembly using long-read shotgun metagenomic sequencing of viruses in fecal and serum samples from marine mammals. Front Microbiol. (2023) 14:1248323. doi: 10.3389/fmicb.2023.1248323 37808316 PMC10556685

[11] Grosser S Sauer J Paijmans AJ Caspers BA Forcada J Wolf JBW. Fur seal microbiota are shaped by the social and physical environment, show mother-offspring similarities and are associated with host genetic quality. Mol Ecol. (2019) 28:2406–22. doi: 10.1111/mec.15070, PMID: 30849214

[12] Kydyrmanov AI Karamendin KO. Viruses of marine mammals and metagenomic monitoring of infectious diseases. Bull Natl Acad Sci Repub Kazakhstan. (2019) 4:147–53. doi: 10.32014/2019.2518-1467.103

[13] Puryear W Sawatzki K Hill N Foss A Stone JJ Doughty L. Highly pathogenic avian influenza a(H5N1) virus outbreak in New England seals, United States. Emerg Infect Dis. (2023) 29:786–91. doi: 10.3201/eid2904.221538, PMID: 36958010 PMC10045683

[14] Ulloa M Fernández A Ariyama N Colom-Rivero A Rivera C Nuñez P. Mass mortality event in south American sea lions (Otaria flavescens) correlated to highly pathogenic avian influenza (HPAI) H5N1 outbreak in Chile. Vet Q. (2023) 43:1–10. doi: 10.1080/01652176.2023.2265173, PMID: 37768676 PMC10588531

[15] Canova R Budaszewski RF Weber MN da Silva MS Puhl DE Battisti LO. Spleen and lung virome analysis of south American fur seals (Arctocephalus australis) collected on the southern Brazilian coast. Infect Genet Evol. (2021) 92:104862. doi: 10.1016/j.meegid.2021.104862, PMID: 33848685

[16] Li L Shan T Wang C Côté C Kolman J Onions D. The fecal viral flora of California Sea lions. J Virol. (2011) 85:9909–17. doi: 10.1128/JVI.05026-11, PMID: 21795334 PMC3196430

[17] Chiappetta CM Cibulski SP Lima FES Varela APM Amorim DB Tavares M. Molecular detection of circovirus and adenovirus in feces of Fur seals (Arctocephalus spp.). EcoHealth. (2017) 14:69–77. doi: 10.1007/s10393-016-1195-8, PMID: 27803979 PMC7087719

[18] Kluge M Campos FS Tavares M de Amorim DB Valdez FP Giongo A. Metagenomic survey of viral diversity obtained from feces of Subantarctic and south American Fur seals. PLoS One. (2016) 11:e0151921. doi: 10.1371/journal.pone.0151921 26986573 PMC4795697

[19] Fahsbender E Burns JM Kim S Kraberger S Frankfurter G Eilers AA. Diverse and highly recombinant anelloviruses associated with Weddell seals in Antarctica. Virus Evol. (2017) 3:vex017. doi: 10.1093/ve/vex017 28744371 PMC5518176

[20] Patterson QM Kraberger S Martin DP Shero MR Beltran RS Kirkham AL. Circoviruses and cycloviruses identified in Weddell seal fecal samples from McMurdo Sound, Antarctica. Infect Genet Evol. (2021) 95:105070. doi: 10.1016/j.meegid.2021.105070, PMID: 34481994 PMC9128802

[21] Regney M Kraberger S Custer JM Crane AE Shero MR Beltran RS. Diverse papillomaviruses identified from Antarctic fur seals, leopard seals and Weddell seals from the Antarctic. Virology. (2024) 594:110064. doi: 10.1016/j.virol.2024.110064, PMID: 38522135

[22] Smeele ZE Burns JM van Doorsaler K Fontenele RS Waits K Stainton D. Diverse papillomaviruses identified in Weddell seals. J Gen Virol. (2018) 99:549–57. doi: 10.1099/jgv.0.001028, PMID: 29469687 PMC5982131

[23] Ng TFF Wheeler E Greig D Waltzek TB Gulland F Breitbart M. Metagenomic identification of a novel anellovirus in Pacific harbor seal (Phoca vitulina richardsii) lung samples and its detection in samples from multiple years. J Gen Virol. (2011) 92:1318–23. doi: 10.1099/vir.0.029678-0, PMID: 21402596

[24] Rosales SM Thurber RV. Brain Meta-Transcriptomics from harbor seals to infer the role of the microbiome and Virome in a stranding event. PLoS One. (2015) 10:e0143944. doi: 10.1371/journal.pone.0143944 26630132 PMC4668051

[25] Bodewes R Contreras GJS García AR Hapsari R van de Bildt M Kuiken T. Identification of DNA sequences that imply a novel gammaherpesvirus in seals. J Gen Virol. (2015) 96:1109–14. doi: 10.1099/vir.0.000029, PMID: 25524165

[26] Goodman SJ. Caspian seal: Pusa caspica. In: Wursig B Thewissen JGM Kovacs K, editors. Encyclopedia of marine mammals. 3rd ed. San Diego, USA: Academic Press (2017). 164–6.

[27] Goodman S Dmitrieva L. Pusa caspica. The IUCN red list of threatened species (2016). e.T41669A45230700. doi: 10.2305/IUCN.UK.2016-1.RLTS.T41669A45230700.en

[28] Vylegzhanin AF. Infectious diseases of fish and seals in the Caspian Sea – ulcerative and "skin blistering" fish disease, diplococcal and vibrio disease of seals. Kharkiv p. (1967):32.

[29] Chuvakova ZK Ikranbegiin R Glebova T. Review of information and results of expeditions to the northern Caspian in connection with the mass death of seals in 2000. Izvestiya NAN RK Ser Biol I Med. (2001) 3:47–54.

[30] Kuiken T Kennedy S Barrett T van de Bildt MWG Borgsteede FH Brew SD. The 2000 canine distemper epidemic in Caspian seals (Phoca caspica): pathology and analysis of contributory factors. Vet Pathol. (2006) 43:321–38. doi: 10.1354/vp.43-3-321, PMID: 16672579

[31] Kennedy S Kuiken T Jepson PD Deaville R Forsyth M Barrett T. Mass die-off of Caspian seals caused by canine distemper virus. Emerg Infect Dis. (2000) 6:637–9. doi: 10.3201/eid0606.000613, PMID: 11076723 PMC2640919

[32] Wilson SC Eybatov TM Amano M Jepson PD Goodman SJ. The role of canine distemper virus and persistent organic pollutants in mortality patterns of Caspian seals (Pusa caspica). PLoS One. (2014) 9:e99265. doi: 10.1371/journal.pone.0099265, PMID: 24987857 PMC4079250

[33] Kydyrmanov A Karamendin K Kassymbekov Y Kumar M Mazkirat S Suleimenova S. Exposure of wild Caspian seals (Pusa caspica) to parasites, bacterial and viral pathogens, evaluated via molecular and serological assays. Front Mar Sci. (2023) 10:10. doi: 10.3389/fmars.2023.1087997

[34] Ivanov VP Kamakin AM Ushivtzev VB Shiganova T Zhukova O Aladin N. Invasion of the Caspian Sea by the comb jellyfish Mnemiopsis Leidyi (Ctenophora). Biol Invasions. (2000) 2:255–8. doi: 10.1023/A:1010098624728

[35] Wilson SC Trukhanova I Dmitrieva L Dolgova E Crawford I Baimukanov M. Assessment of impacts and potential mitigation for icebreaking vessels transiting pupping areas of an ice-breeding seal. Biol Conserv. (2017) 214:213–22. doi: 10.1016/j.biocon.2017.05.028

[36] Dmitrieva L Kondakov AA Oleynikov E Kydyrmanov A Karamendin K Kasimbekov Y. Assessment of Caspian seal by-catch in an illegal fishery using an interview-based approach. PLoS One. (2013) 8:e67074. doi: 10.1371/journal.pone.0067074, PMID: 23840590 PMC3694144

[37] Harkonen T Harding KC Wilson S Baimukanov M Dmitrieva L Svensson CJ. Collapse of a marine mammal species driven by human impacts. PLoS One. (2012) 7:e43130. doi: 10.1371/journal.pone.0043130, PMID: 23028446 PMC3446954

[38] Georgieva M. Investigation underway into cause of hundreds of dead seals in Caspian Sea. (2020); The corpses of the endangered mammals were found on the shores of Russia's Dagestan region. Available at: https://www.telegraph.co.uk/news/2020/12/14/investigation-underway-cause-hundreds-dead-seals-caspian-sea/ (accessed May, 2024).

[39] Anonymous. Marine Mammal Commission. The marine mammal protection act of 1972 as amended 2018. National Marine Mammal Tissue Bank and tissue analysis. (2019); Available at: https://www.mmc.gov/wp-content/uploads/MMPA_March2019.pdf (Accessed September 03, 2024).

[40] Andrews S., A quality control tool for high throughput sequence data (2010). Available at: http://www.bioinformatics.babraham.ac.uk/projects/fastqc/ (Accessed September 03, 2024).

[41] Kearse M Moir R Wilson A Stones-Havas S Cheung M Sturrock S. Geneious basic: an integrated and extendable desktop software platform for the organization and analysis of sequence data. Bioinformatics. (2012) 28:1647–9. doi: 10.1093/bioinformatics/bts199, PMID: 22543367 PMC3371832

[42] Prjibelski A Antipov D Meleshko D Lapidus A Korobeynikov A. Using SPAdes De novo assembler. Curr Protoc Bioinformatics. (2020) 70:e102. doi: 10.1002/cpbi.102 32559359

[43] Carissimo G van den Beek M Vernick KD Antoniewski C. Metavisitor, a suite of galaxy tools for simple and rapid detection and discovery of viruses in deep sequence data. PLoS One. (2017) 12:e0168397. doi: 10.1371/journal.pone.0168397, PMID: 28045932 PMC5207757

[44] Barrett T Visser IKG Mamaev L Goatley L van Bressem MF Osterhaus ADME. Dolphin and porpoise morbilliviruses are genetically distinct from phocine distemper virus. Virology. (1993) 193:1010–2. doi: 10.1006/viro.1993.1217 8460473

[45] Goldstein T Colegrove KM Hanson M Gulland FMD. Isolation of a novel adenovirus from California Sea lions. Dis Aquat Org. (2011) 94:243–8. doi: 10.3354/dao02321 21790072

[46] Harder TC Vos H de Swart RL Osterhaus ADME. Age-related disease in recurrent outbreaks of phocid herpesvirus type-1 infections in a seal rehabilitation Centre: evaluation of diagnostic methods. Vet Rec. (1997) 140:500–3. doi: 10.1136/vr.140.19.500, PMID: 9172297

[47] Osterhaus ADME Rimmelzwaan GF Martina BEE Bestebroer TM Fouchier RAM. Influenza B virus in seals. Science. (2000) 288:1051–3. doi: 10.1126/science.288.5468.1051, PMID: 10807575

[48] Payungporn S Phakdeewirot P Chutinimitkul S Theamboonlers A Keawcharoen J Oraveerakul K. Single-step multiplex reverse transcription-polymerase chain reaction (RT-PCR) for influenza a virus subtype H5N1 detection. Viral Immunol. (2004) 17:588–93. doi: 10.1089/vim.2004.17.588, PMID: 15671756

[49] Kumar S Stecher G Li M Knyaz C Tamura K. MEGA X: molecular evolutionary genetics analysis across computing platforms. Mol Biol Evol. (2018) 35:1547–9. doi: 10.1093/molbev/msy096, PMID: 29722887 PMC5967553

[50] Gu Z Eils R Schlesner M. Complex heatmaps reveal patterns and correlations in multidimensional genomic data. Bioinformatics. (2016) 32:2847–9. doi: 10.1093/bioinformatics/btw313 27207943

[51] Geoghegan JL Pirotta V Harvey E Smith A Buchmann JP Ostrowski M. Virological sampling of inaccessible wildlife with drones. Viruses. (2018) 10:300. doi: 10.3390/v10060300, PMID: 29865228 PMC6024715

[52] Jackson EW Wilhelm RC Johnson MR Lutz HL Danforth I Gaydos JK. Diversity of sea star-associated Densoviruses and transcribed endogenous viral elements of Densovirus origin. J Virol. (2021) 95:e01594-20. doi: 10.1128/JVI.01594-20 PMC7737747 32967964

[53] Reid SM King DP Shaw AE Knowles NJ Hutchings GH Cooper EJ. Development of a real-time reverse transcription polymerase chain reaction assay for detection of marine caliciviruses (genus Vesivirus). J Virol Methods. (2007) 140:166–73. doi: 10.1016/j.jviromet.2006.11.010, PMID: 17187870

[54] de la Higuera I Kasun GW Torrance EL Pratt AA Maluenda A Colombet J. Unveiling Crucivirus diversity by mining metagenomic data. MBio. (2020) 11:e01410-20. doi: 10.1128/mBio.01410-20 32873755 PMC7468197

[55] Meng X-J. Circoviridae. In: Knipe DM Howley PM, editors. Fields virology. Philadelphia, PA: Wolters Kluwer/Lippincott Williams & Wilkins Health (2013). 2664.

[56] Rosario K Schenck RO Harbeitner RC Lawler SN Breitbart M. Novel circular single-stranded DNA viruses identified in marine invertebrates reveal high sequence diversity and consistent predicted intrinsic disorder patterns within putative structural proteins. Front Microbiol. (2015) 6:696. doi: 10.3389/fmicb.2015.00696 26217327 PMC4498126

[57] Osterhaus AD Yang H Spijkers HE Groen J Teppema JS Van Steenis G. The isolation and partial characterization of a highly pathogenic herpesvirus from the harbor seal (Phoca vitulina). Arch Virol. (1985) 86:239–51. doi: 10.1007/BF01309828, PMID: 4062560

[58] Gulland FM Lowenstine LJ Lapointe JM Spraker T King DP. Herpesvirus infection in stranded Pacific harbor seals of coastal California. J Wildl Dis. (1997) 33:450–8. doi: 10.7589/0090-3558-33.3.450, PMID: 9249689

[59] Wellehan JF Cortes-Hinojosa G. Marine mammal viruses. In: Miller RE Lamberski N Calle P, editors. Fowler's zoo and wild animal medicine: current therapy. Philadelphia: Saunders (2018)

[60] Van Bressem MF Van Waerebeek K Raga JA. A review of virus infections of cetaceans and the potential impact of morbilliviruses, poxviruses and papillomaviruses on host population dynamics. Dis Aquat Org. (1999) 38:53–65. 10.3354/dao038053 10590929

[61] Ghim SJ Joh J Mignucci-Giannoni AA Rivera-Guzmán AL Falcón-Matos L Alsina-Guerrero MM. Genital papillomatosis associated with two novel Mucosotropic papillomaviruses from a Florida manatee (Trichechus manatus latirostris). Aquat Mamm. (2014) 40:195–200. doi: 10.1578/AM.40.2.2014.195

[62] Robles-Sikisaka R Rivera R Nollens HH St Leger J Durden WN Stolen M. Evidence of recombination and positive selection in cetacean papillomaviruses. Virology. (2012) 427:189–97. doi: 10.1016/j.virol.2012.01.039 22386054

[63] Rivera R Robles-Sikisaka R Hoffman EM Stacy BA Jensen ED Nollens HH. Characterization of a novel papillomavirus species (ZcPV1) from two California Sea lions (Zalophus californianus). Vet Microbiol. (2012) 155:257–66. doi: 10.1016/j.vetmic.2011.09.027, PMID: 22005176

[64] He WT Hou X Zhao J Sun J He H Si W. Virome characterization of game animals in China reveals a spectrum of emerging pathogens. Cell. (2022) 185:1117. doi: 10.1016/j.cell.2022.02.014 35298912 PMC9942426

[65] Anthony SJ St Leger JA Liang E Hicks AL Sanchez-Leon MD Jain K. Discovery of a novel Hepatovirus (Phopivirus of seals) related to human hepatitis a virus. MBio. (2015) 6:e01180-15. doi: 10.1128/mBio.01180-15, PMID: 26307166 PMC4550696

[66] Rodrigues TCS Nielsen O Burek-Huntington KA Popov VL Raverty S Lambourn DM. Genomic characterization of picornaviruses isolated from ribbon (Histriophoca fasciata) and harbor (Phoca vitulina) seals. Front Vet Sci. (2020) 7:554716. doi: 10.3389/fvets.2020.554716, PMID: 33195526 PMC7661754

[67] Green KY Ando T Balayan MS Berke T Clarke IN Estes MK. Taxonomy of the caliciviruses. J Infect Dis. (2000) 181:S322–30. doi: 10.1086/315591 10804145

[68] Smith AW Akers TG Madin SH Vedros NA. San Miguel Sea lion virus isolation, preliminary characterization and relationship to vesicular exanthema of swine virus. Nature. (1973) 244:108–10. doi: 10.1038/244108a0, PMID: 4583480

[69] Kaczorowska J van der Hoek L. Human anelloviruses: diverse, omnipresent and commensal members of the virome. FEMS Microbiol Rev. (2020) 44:305–13. doi: 10.1093/femsre/fuaa007 32188999 PMC7326371

[70] Kyathanahalli C Snedden M Hirsch E. Human Anelloviruses: prevalence and clinical significance during pregnancy. Front Virol. (2021) 1:1. doi: 10.3389/fviro.2021.782886

[71] Webster RG Hinshaw VS Bean WJ van Wyke KL Geraci JR St Aubin DJ. Characterization of an influenza-a virus from seals. Virology. (1981) 113:712–24. doi: 10.1016/0042-6822(81)90200-2, PMID: 6267805

[72] Hinshaw VS Bean WJ Webster RG Rehg JE Fiorelli P Early G. Are seals frequently infected with avian influenza-viruses. J Virol. (1984) 51:863–5. doi: 10.1128/jvi.51.3.863-865.1984, PMID: 6471169 PMC255856

[73] Callan RJ Early G Kida H Hinshaw VS. The appearance of H3 influenza-viruses in seals. J Gen Virol. (1995) 76:199–203. doi: 10.1099/0022-1317-76-1-199, PMID: 7844533

[74] Hinshaw VS Bean WJ Geraci J Fiorelli P Early G Webster RG. Characterization of two influenza a viruses from a pilot whale. J Virol. (1986) 58:655–6. doi: 10.1128/jvi.58.2.655-656.1986, PMID: 3701925 PMC252957

[75] Goldstein T Mena I Anthony SJ Medina R Robinson PW Greig DJ. Pandemic H1N1 influenza isolated from free-ranging northern elephant seals in 2010 off the Central California coast. PLoS One. (2013) 8:e62259. doi: 10.1371/journal.pone.0062259, PMID: 23690933 PMC3655164

[76] Lair S Quesnel L Signore AV Delnatte P Embury-Hyatt C Nadeau MS. Outbreak of highly pathogenic avian influenza a(H5N1) virus in seals, St. Lawrence estuary, Quebec, Canada. Emerg Infect Dis. (2024) 30:1133–43. doi: 10.3201/eid3006.231033 38781927 PMC11138997

[77] Campagna C Uhart M Falabella V Campagna J Zavattieri V Vanstreels RET. Catastrophic mortality of southern elephant seals caused by H5N1 avian influenza. Mar Mamm Sci. (2024) 40:322–5. doi: 10.1111/mms.13101

[78] Bennison A Byrne AM Reid SM Lynton-Jenkins JG Mollett B Silva DD. Detection and spread of high pathogenicity avian influenza virus H5N1 in the Antarctic region. bioRxiv. (2024):2023.11.23.568045. doi: 10.1101/2023.11.23.568045 PMC11372179 39227574

[79] Gulyaeva M Sobolev I Sharshov K Kurskaya O Alekseev A Shestopalova L. Characterization of avian-like influenza a (H4N6) virus isolated from Caspian seal in 2012. Virol Sin. (2018) 33:449–52. doi: 10.1007/s12250-018-0053-y, PMID: 30328579 PMC6235759

[80] Ohishi K Ninomiya A Kida H Park CH Maruyama T Arai T. Serological evidence of transmission of human influenza a and B viruses to Caspian seals (Phoca caspica). Microbiol Immunol. (2002) 46:639–44. doi: 10.1111/j.1348-0421.2002.tb02746.x, PMID: 12437032

[81] Gadzhiev A Petherbridge G Sharshov K Sobolev I Alekseev A Gulyaeva M. *Pinnipeds and avian influenza: a global timeline and review of research on the impact of highly pathogenic avian influenza on pinniped populations with particular reference to the endangered Caspian seal (Pusa caspica).* Frontiers in cellular and infection. Microbiology. (2024) 14:14. doi: 10.3389/fcimb.2024.1325977 PMC11273096 39071164

[82] Sludskiy A Badamshin B Bekenov A Grachev Y Kydyrbayev H Lazarev A. Mammals of Kazakhstan. In: Gvozdev EV Strautman EI, editors. Carnivora (Canidae, Ursidae, Procyonidae), and Pinnipedia (Phocidae) (in Russian). Alma-Ata, Kazakhstan: Nauka of Kazakh SSR (1985). 244.

[83] Gamarra-Toledo V Plaza PI Gutiérrez R Inga-Diaz G Saravia-Guevara P Pereyra-Meza O. Mass mortality of sea lions caused by highly pathogenic avian influenza a(H5N1) virus. Emerg Infect Dis. (2023) 29:2553–6. doi: 10.3201/eid2912.230192, PMID: 37916983 PMC10683807

[84] Bodewes R Morick D de Mutsert G Osinga N Bestebroer T van der Vliet S. Recurring influenza B virus infections in seals. Emerg Infect Dis. (2013) 19:511–2. doi: 10.3201/eid1903.120965, PMID: 23750359 PMC3647654

[85] Anindita PD Sasaki M Gonzalez G Phongphaew W Carr M Hang’ombe BM. Discovery and genetic characterization of diverse smacoviruses in Zambian non-human primates. Sci Rep. (2019) 9:5045. doi: 10.1038/s41598-019-41358-z, PMID: 30962460 PMC6453971

[86] Quaiser A Krupovic M Dufresne A Francez AJ Roux S. Diversity and comparative genomics of chimeric viruses in Sphagnum-dominated peatlands. Virus Evol. (2016) 2:vew025. doi: 10.1093/ve/vew025 29492276 PMC5822885

[87] Evermann JF Kennedy MA. Chapter 16- viral infections. In: Peterson ME Kutzler MA, editors. Small Animal Pediatrics. Saint Louis: W.B. Saunders (2011). 119–29.

[88] Inoshima Y Murakami T Ishiguro N Hasegawa K Kasamatsu M. An outbreak of lethal adenovirus infection among different otariid species. Vet Microbiol. (2013) 165:455–9. doi: 10.1016/j.vetmic.2013.04.013, PMID: 23643878

[89] Wright EP Waugh LF Goldstein T Freeman KS Kelly TR Wheeler EA. Evaluation of viruses and their association with ocular lesions in pinnipeds in rehabilitation. Vet Ophthalmol. (2015) 18:148–59. doi: 10.1111/vop.12235, PMID: 25400019

[90] Cortes-Hinojosa G Doescher B Kinsel M Lednicky J Loeb J Waltzek T. Coinfection of California Sea lion adenovirus 1 and a novel polyomavirus in a Hawaiian monk seal (Neomonachus Schauinslandi). J Zoo Wildl Med. (2016) 47:427–37. doi: 10.1638/2014-0252.1, PMID: 27468013

[91] Grachev MA Kumarev VP Mamaev LV Zorin VL Baranova LV Denikina NN. Distemper virus in Baikal seals. Nature. (1989) 338:209–10. doi: 10.1038/338209a0 2922047

[92] Namroodi S Shirazi AS Khaleghi SR Mills JN Kheirabady V. Frequency of exposure of endangered Caspian seals to canine distemper virus, leptospira interrogans, and toxoplasma gondii. PLoS One. (2018) 13:e0196070. doi: 10.1371/journal.pone.0196070, PMID: 29698496 PMC5919510

[93] Wylie KM Mihindukulasuriya KA Zhou Y Sodergren E Storch GA Weinstock GM. Metagenomic analysis of double-stranded DNA viruses in healthy adults. BMC Biol. (2014) 12:71. doi: 10.1186/s12915-014-0071-7, PMID: 25212266 PMC4177058

[94] Hilgen FJ Lourens LJ Van Dam JA Beu AG Boyes AF Cooper RA. Chapter 29 - The Neogene Period. In: Gradstein FM Ogg JG Schmitz MD Ogg GM, editors The Geologic Time Scale. (2012), Vols 1 & 2, Elsevier p. 923–978.

[95] Krijgsman W Tesakov A Yanina T Lazarev S Danukalova G van Baak CGC. Quaternary time scales for the Pontocaspian domain: Interbasinal connectivity and faunal evolution. Earth Sci Rev. (2019) 188:1–40. doi: 10.1016/j.earscirev.2018.10.013

[96] Nyakatura K Bininda-Emonds ORP. Updating the evolutionary history of Carnivora (Mammalia): a new species-level supertree complete with divergence time estimates. BMC Biol. (2012) 10:10. doi: 10.1186/1741-7007-10-12 22369503 PMC3307490

