# Supplementary material for: Viral metagenomic survey of Caspian seals
Source: Front Vet Sci. 2024 Sep 18;11:1461135. doi: 10.3389/fvets.2024.1461135 (PMC11445147; doi:10.3389/fvets.2024.1461135)
Supplement: Supplementary file 3 [file Table_2.docx]

Genbank Accession numbers used in the article

| **Accession #** | **Virus Contig/Strain** | **Link to Genbank** |
| --- | --- | --- |
| PP744471 | Circoviridae | <https://www.ncbi.nlm.nih.gov/nucleotide/PP744471> |
| PP744472 | Parvoviridae | <https://www.ncbi.nlm.nih.gov/nucleotide/PP744472> |
| PP744473 | Parvoviridae | <https://www.ncbi.nlm.nih.gov/nucleotide/PP744473> |
| PP744474 | Herpesviridae | <https://www.ncbi.nlm.nih.gov/nucleotide/PP744474> |
| PP744475 | Papillomaviridae | <https://www.ncbi.nlm.nih.gov/nucleotide/PP744475> |
| PP744476 | Picornaviridae | <https://www.ncbi.nlm.nih.gov/nucleotide/PP744476> |
| PP744477 | Caliciviridae | <https://www.ncbi.nlm.nih.gov/nucleotide/PP744477> |
| PP744478 | Anelloviridae | <https://www.ncbi.nlm.nih.gov/nucleotide/PP744478> |
| PP744479 | Smacoviridae | <https://www.ncbi.nlm.nih.gov/nucleotide/PP744479> |
| PP744480 | Orthomyxoviridae/ Influenza A | <https://www.ncbi.nlm.nih.gov/nucleotide/PP744480> |
| PP744481 | Orthomyxoviridae/ Influenza A | <https://www.ncbi.nlm.nih.gov/nucleotide/PP744481> |
| PP744482 | Orthomyxoviridae/ Influenza B | <https://www.ncbi.nlm.nih.gov/nucleotide/PP744482> |
| PP744483 | Orthomyxoviridae/ Influenza B | <https://www.ncbi.nlm.nih.gov/nucleotide/PP744483> |
| AWR89666 | Replication initiation protein [Humpback whale blow-associated circo-like virus 2] | <https://www.ncbi.nlm.nih.gov/nucleotide/AWR89666> |
| QOD39598 | NS2 protein [uncultured sea star-associated densovirus] | <https://www.ncbi.nlm.nih.gov/nucleotide/QOD39598> |
| QBO24456 | capsid protein VP1 [Canine parvovirus] | <https://www.ncbi.nlm.nih.gov/nucleotide/QBO24456> |
| AII80971 | protein EUL25 [Equid alphaherpesvirus 1] | <https://www.ncbi.nlm.nih.gov/nucleotide/AII80971> |
| NC_040818 | E2 protein [Leptonychotes weddellii papillomavirus 6] | <https://www.ncbi.nlm.nih.gov/nucleotide/NC_040818> |
| UMO75562 | Polyprotein [Racoon dog picornavirus] | <https://www.ncbi.nlm.nih.gov/nucleotide/UMO75562> |
| AKG26793 | ORF-1 protein [San Miguel sea lion virus 8] | <https://www.ncbi.nlm.nih.gov/nucleotide/AKG26793> |
| YP_009115496.1 | ORF1 [Seal anellovirus 4] | <https://www.ncbi.nlm.nih.gov/nucleotide/YP_009115496.1> |
| QCC72666 | capsid protein [Swine associated smacovirus] | <https://www.ncbi.nlm.nih.gov/nucleotide/QCC72666> |
| QKV51008 | putative replication associated protein [Crucivirus sp.] | <https://www.ncbi.nlm.nih.gov/nucleotide/QKV51008> |
| MT406985 | hemagglutinin gene (Influenza A virus A/Anas platyrhynchos/Belgium/3950-8/2015(H3N8)) | <https://www.ncbi.nlm.nih.gov/nucleotide/MT406985> |
| QDX48107 | nonstructural protein 1 (NS1) gene [Influenza A/Blue-winged Teal/Kansas/AH0029699S.8.B/2015 (H12N6)] | <https://www.ncbi.nlm.nih.gov/nucleotide/QDX48107> |
| APW80077 | polymerase PB2 [Influenza B virus (B/Florida/09/2016)] | <https://www.ncbi.nlm.nih.gov/nucleotide/APW80077> |
| AQS98133 | NS protein [Influenza B virus (B/Arizona/38/2016)] | <https://www.ncbi.nlm.nih.gov/nucleotide/AQS98133> |
